# Sublethal pesticide doses negatively affect survival and the cellular responses in American foulbrood-infected honeybee larvae

**DOI:** 10.1038/srep40853

**Published:** 2017-02-01

**Authors:** Javier Hernández López, Sophie Krainer, Antonia Engert, Wolfgang Schuehly, Ulrike Riessberger-Gallé, Karl Crailsheim

**Affiliations:** 1 Institute of Zoology, Universitätsplatz 2, University of Graz, A-8010 Graz, Austria

## Abstract

Disclosing interactions between pesticides and bee infections is of most interest to understand challenges that pollinators are facing and to which extent bee health is compromised. Here, we address the individual and combined effect that three different pesticides (dimethoate, clothianidin and fluvalinate) *and* an American foulbrood (AFB) infection have on mortality and the cellular immune response of honeybee larvae. We demonstrate for the first time a synergistic interaction when larvae are exposed to sublethal doses of dimethoate or clothianidin in combination with *Paenibacillus larvae*, the causative agent of AFB. A significantly higher mortality than the expected sum of the effects of each individual stressor was observed in co-exposed larvae, which was in parallel with a drastic reduction of the total and differential hemocyte counts. Our results underline that characterizing the cellular response of larvae to individual and combined stressors allows unmasking previously undetected sublethal effects of pesticides in colony health.

During the last decade numerous investigations have reported declines in abundance and diversity of bee populations due to multiple factors such as the spread of parasites and pathogens, the use of pesticides for crop management and a reduction in the availability of natural habitats^1^,^2^,^3^,^4^,^5^. Undoubtedly, the worldwide generalized use of insecticides such as neonicotinoids has contributed and exacerbated the rapid losses reported for bees^6^,^7^,^8^,^9^. Numerous examples of sublethal effects of pesticides on behavior, foraging activity, learning performance, reproduction or immunity can be found in the literature^10^,^11^,^12^,^13^,^14^,^15^,^16^,^17^. It becomes more and more evident that stressors, e.g. insecticides and bee pathogens can interact together in a synergistic manner^18^,^19^,^20^,^21^. There are also reported cases of synergistic interactions between parasite and pathogen or between chemicals^21^,^22^,^23^,^24^,^25^.

The estimation of the dietary risk that honeybee larvae face with regard to pesticides is a complex matter. To do so, we firstly need to consider the field realistic concentrations of pesticides present in honey, pollen and bee-products like royal jelly and wax. Insecticides have been applied to crops for several decades now and they may possess half-lives of up to several years^6^,^26^,^27^. Moreover, neonicotinoids are broken down in the environment or metabolized in organisms into several metabolites, some of them were identified to possess similar or even greater toxicity than the untransformed compound^28^,^29^,^30^. Analyses of thiamethoxam residues in pollen of coated sunflower seeds revealed presence in only one sample. However clothianidin, which is one of the degradation metabolites of thiamethoxam, yielded concentrations ranging from 40–58 ng/g in all samples analyzed^29^. Overall, literature is reporting significantly higher concentrations of pesticide residues in most recent studies. This is likely due to the accumulation of pesticides in the environment after years of application, better detection techniques with higher recovery rates and the inclusion of metabolites in the analyses^27^,^31^,^32^. Thus, average values of pesticide residues as a mean of the different concentrations found and published from investigations carried out during the last 10–15 years seem to underestimate the real concentrations of pesticides currently prevailing^7^,^33^,^34^,^35^,^36^. For instance, clothianidin, which accounted for over 50% of the total pollen collected, was found up to 88 ng/g in maize pollen and up to 13.3 ng/g in dead bees from colonies placed near treated crops^35^.

The application techniques of pesticides used to treat crops generate significant differences in residue concentrations^37^,^38^. Here, neonicotinoid metabolites accounted for 15.5–27.2% of the total residue amounts found, which yielded average levels up to 122 ng/g in pollen and 17.6 ng/g in nectar^37^.

Secondly, residue analyses in individual samples of pollen, honey or bee wax revealed the presence of a cocktail of multiple insecticides accumulating at the same time^34^,^35^,^39^,^40^,^41^. It was also demonstrated that rearing brood in such contaminated combs causes a delayed development of larvae and emergence as well as a shortened adult life-span^42^. The latter authors found an average of 10 different chemicals in the wax, the most frequently detected were fluvalinate (average 6712 ng/g) and coumaphos (average 8079 ng/g), but neonicotinoids such as clothianidin (average 35 ng/g) and thiamethoxam (average 38 ng/g) were also found.

Finally, the dietary risk posed by pesticides is highly dependent on the food consumption by larvae. Honeybee larvae are fed exclusively on royal jelly during the first three days of their development. Thereafter, the diet regime shifts to mostly pollen and honey/nectar for worker larvae, whereas queen larvae receive only royal jelly during their entire development^43^. About 142 mg of honey are needed for the development of one worker larva and a total of 125–187.5 mg of pollen per larva are processed in the hypopharyngeal glands (HPGs) of nurses before feeding^43^,^44^. Rortais *et al*.^45^ reviewed the estimated amounts of sugar and pollen that are consumed by different castes of bees, which were later used by other investigators to estimate dietary exposure risk of bees to insecticides^32^. The authors claimed that a honeybee larva consumes 5.4 mg of pollen during its development but a closer look at the real pollen consumption is needed to assess possible pesticide accumulation. From the total of 125–187.5 mg of pollen needed for the development of one larva, more than 95% is at first consumed and processed in the HPGs into royal jelly by nurse bees, and less than 5% (this is the 5.4 mg that Rortais *et al*. accounted) is fed directly to the larvae. Solely accounting these 5% of the total pollen results in an underestimation of the real pesticide exposure that honeybee larvae face. Especially when we consider the rather monofloral pollen collected in the vicinity of sunflower, maize and oilseed rape crops, which accounts for 60 to 90% of the total pollen collected^7^,^35^,^46^. To the best of our knowledge, nothing is known to date about the passage and modification of chemicals in HPGs and mandibular glands of nurse bees during pollen processing prior to the feeding of larvae or queens.

The amount of sugar that honeybee larvae consume also influences the dietary exposure of larvae to pesticides. It has been calculated that about 142 mg of honey are needed for the development of one larva^43^,^45^. The sugar content in honey is *ca* 80%, but in nectar it varies tremendously depending on the floral origin, with an average content of 10–40%^47^, an average of 40% in the nectar of sunflower plants^48^ and about 28–42% in *Cucurbita pepo*^49^. This means that several hundred μl of nectar are needed to feed one larva (or produce 142 mg of honey) depending on the nectar origin.

Based on these arguments, it seems plausible that the dietary exposure of honeybee larvae is even higher than estimated until now. Sanchez-Bayo *et al*.^32^ developed an approach to determine the risks by “cumulative toxicity”, applied to insecticides that exhibit time-cumulative toxicity due to binding to specific receptors. Under this approach, and taking into account that mean levels of insecticides found for several years were used, larvae fed with thiamethoxam-contaminated pollen would reach the worker LD_50_ (5 ng) in less than one day.

Here we tested the hypothesis whether exposure to a sublethal dose of dimethoate or clothianidin or the ~LD_30_ of fluvalinate renders honeybee larvae more susceptible to the Gram+ bacteria *Paenibacillus larvae (P. larvae*), causative agent of American Foulbrood (AFB). AFB is considered to be the most threatening bacterial disease of honeybee brood. The spores represent *P. larvae*’s infectious stage and adult honeybees, which are resistant to infection, serve as vectors within and between colonies, delivering spores to the brood while nursing^50^. During experimentation, larvae were artificially reared and exposed to insecticides, *P. larvae* spores or a combination of both, then mortality was recorded daily and the cellular response was evaluated by assessing total hemocyte counts (THC) and differential hemocyte counts (DHC).

## Results

### Individual effect of pesticides or bacterial infection on larval mortality

**Figure 1:**
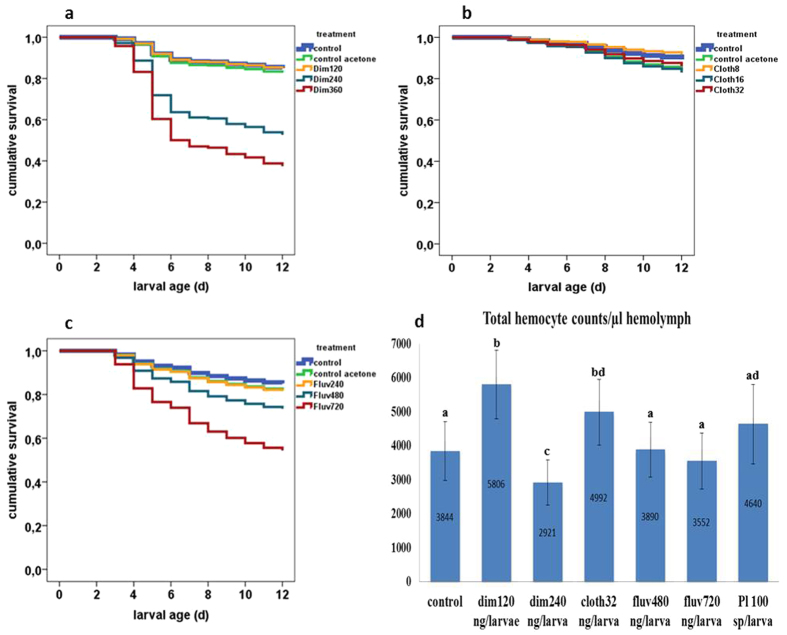
Effects of (**a**) dimethoate, (**b**) clothianidin, and (**c**) fluvalinate on larval mortality; control; n = 384, control acetone; n = 288, dim120; n = 194, dim240; n = 144, dim360; n = 144, cloth8; n = 120, cloth16; n = 120, cloth32; n = 144, fluv240; n = 144, fluv480; n = 144, fluv720; n = 144. (**d**) Effect of dimethoate, clothianidin, fluvalinate or *P. larvae* spores on the no. of total hemocytes counts (THC) in honey bee larvae. Control: n = 34; dim120: n = 35; dim240: n = 23; cloth32: n = 33; fluv480: n = 32; fluv720: n = 22; *P. larvae* sp: n = 30. Error bars represent standard deviation.

A Cox regression analysis showed significant differences in the mortality rate of larvae according to treatments (see Table 1 for statistics, Fig. 1a–c for survival curves). Application of 240 and 360 ng/larva of dimethoate caused 47.2% (~LD_50_) and 63.9% larval mortality, respectively. Application of 240, 460 and 720 ng/larva of fluvalinate resulted in 20.8%, 29.9% (~LD_30_) and 48.6% (~LD_50_) larval mortality, respectively. When feeding 120 ng/larva of dimethoate or 8, 16 and 32 ng/larva of clothianidin no significant differences compared to control were observed during the 12d of experimentation and such doses were considered as sublethal for subsequent experiments. Infection of larvae with *ca* 100 *P. larvae* spores produced 45.2% of larval mortality.

### Individual effect of pesticides or bacterial infection on hemocyte counts

**Figure 2:**
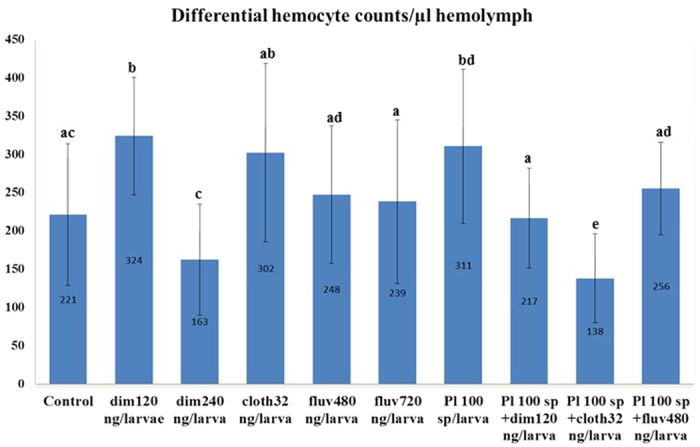
Changes in the no. of differential hemocytes counts (DHC) regarding feeding regime of honeybee larvae. Control: n = 34; dim120: n = 35; dim240: n = 23; cloth32: n = 33; fluv480: n = 32; fluv720: n = 22; *P. larvae* sp: n = 30; *P. larvae* sp + dim120: n = 31; *P. larvae* sp + cloth32: n = 30; *P. larvae* sp + fluv480: n = 32. Error bars represent standard deviation.

Statistically significant differences for THC and DHC were found among the different experimental groups. When the organophosphate dimethoate was fed at a sublethal dose (dim120 ng/larva), an increase of the THC and DHC was observed as compared to control (see Fig. 1d for THC, Fig. 2 for DHC, Table 2 for statistics). Among differential hemocytes, levels of plasmatocytes did not differ between control and dim120, but levels of granulocytes and oenocytoids were significantly higher (see S. Fig. 1). When dimethoate was fed at the LD_50_ (dim240 ng/larva), a significant reduction on the THC was found as compared to control. No significant difference was observed for DHC between control and dim240. Whereas a sublethal dose of dimethoate(120 ng/larva) activates the cellular response and increases THC and DHC, a dose close to LD_50_ produces a drastic reduction on the hemocyte counts.

When the neonicotinoid clothianidin was fed at a sublethal dose (cloth32 ng/larva) an activation of the cellular response was initiated and an increase of the THC was observed as compared to control. No significant difference was observed for DHC between control larvae and cloth32 (see Fig. 1d for THC, Fig. 2 for DHC, Table 2 for statistics). Nevertheless, levels of granulocytes and oenocytoids were significantly higher in larvae feeding on clothianidin compared to controls (see S. Fig. 1).

When the pyrethroid fluvalinate was fed at two partly lethal concentrations (fluv480 = LD_30_ and fluv720 = LD_50_) no statistically significant differences were found either for THC or DHC at both concentrations as compared to control (see Fig. 1d for THC, Fig. 2 for DHC, Table 2 for statistics). Based on these results, we assume that fluvalinate has none to little effect on the general cellular response. Intriguingly, levels of oenocytoids were significantly higher in larvae feeding on fluv480 and fluv720 compared to control (see S. Fig. 1).

Finally, infection with *P. larvae* spores had no effect on THC when compared to control larvae, but levels of DHC were significantly increased (see Fig. 1d for THC, Fig. 2 for DHC, Table 2 for statistics). This is consistent with previous results where an immune challenge of honeybee queens triggered an increase of DHC in larvae^51^. Similar to dim120 and cloth32 groups, among the types of differential hemocytes, levels of granulocytes and oenocytoids were significantly higher in larvae fed *P. larvae* spores compared to controls (see S. Fig. 1).

### Combined effect of pesticides and bacterial infection on larval mortality and hemocyte counts

Hemocyte counting was performed on d7 on fully developed larvae that did not show weight differences versus control naïve larvae and presented no symptoms of infection and/or intoxication. Alive larvae that did not develop as well as control naïve larvae and that had a dark-brownish appearance are moribund and will die during the following hours to days, their immune defense mechanisms have been overwhelmed and none to very low levels of hemocytes were found (data not shown). By comparing the effect of a combined treatment on the cellular responses (*P. larvae* spores and pesticide exposure) in fully developed and asymptomatic larvae as compared to the effect that each treatment has individually, we can estimate whether larvae rely on hemocytes to fight both stressors.

### Combined effects of dimethoate and *P. larvae* spores

**Figure 3:**
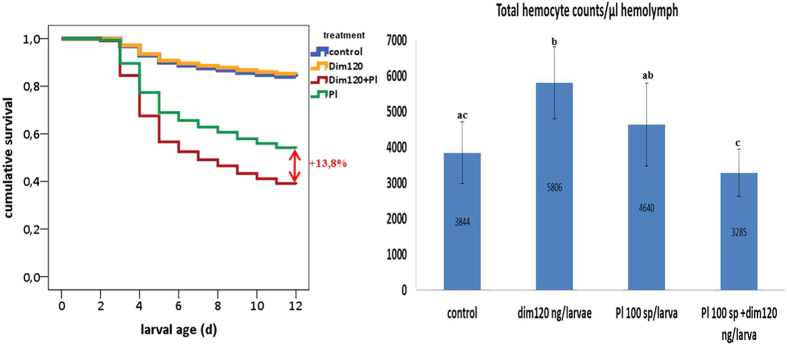
Effects of dimethoate and *P. larvae* on larval mortality; control: n = 144; dim120: n = 194; *P. larvae* sp: n = 239; *P. larvae* sp + dim120: n = 144; and on THC; control: n = 34; dim120: n = 35; *P. larvae* sp: n = 30; *P. larvae* sp + dim120: n = 31 (*). Notice the bias caused by the selection of only fully developed and asymptomatic surviving larvae in all groups for hemocyte counts. Hemocyte levels of larvae with symptoms of infection and/or intoxication are not shown here. Error bars represent standard deviation.

A Cox regression analysis showed significant differences in the mortality rate of larvae regarding treatment (see Table 3 for statistics, Fig. 3 for survival curves). Larval mortality in the control group was 16.7% at the end of the experiment (d12). Feeding a sublethal dose of dim resulted in 14.6% larval mortality and feeding of *P. larvae* spores resulted in 45.2% mortality (results previously presented). When larvae were co-exposed to both stressors, a significantly higher mortality than the sum of the individual effect was observed, resulting in 59.0% mortality at d12, indicating a synergistic effect between dimethoate and a bacterial infection. Similarly, THC and DHC are also affected by the combination of both stressors (see Fig. 3 for THC, Fig. 2 for DHC, Table 2 for statistics). Feeding dim120 to larvae produces an increase in THC and DHC when compared to control and feeding *P. larvae* spores leads to an increase in DHC when compared to naïve larvae (results previously presented). Both stressors applied individually increase the cellular response but when comparing this effect against the effect they cause together in the cellular response, a statistically significant reduction of THC and DHC is found in larvae fed on *P. larvae* spores + dim120 compared to larvae fed on dim120 or *P. larvae* spores solely. Among differential hemocytes, levels of plasmatocytes did not differ significantly in larvae fed on *P. larvae* spores + dim120 compared to levels in larvae fed on *P. larvae* spores or dim120. Nevertheless, levels of granulocytes were significantly lower in larvae fed on *P. larvae* spores + dim120. Taken together, these results suggest that exposure of larvae to a sublethal dose of dimethoate and *P. larvae* spores in combination adds an additional 13.8% to the mortality caused by *P. larvae* spores solely due to a synergistic effect (this is the difference between the mortality caused by *P. larvae* and *P. larvae* + dim120 after subtracting control mortality) and this correlates with a significant immune depletion of the cellular response in the same larvae. It is worth mentioning that these are hemocyte levels measured in fully developed larvae as in alive but moribund, i.e., larvae showing symptoms of infection and/or intoxication due to an overwhelmed immune system, none to very low levels of hemocytes can generally be found (data not shown). The latter larvae were therefore excluded from the hemocyte assessment (exclusion of moribund larvae was followed in all three combination treatments).

### Combined effects of clothianidin and *P. larvae* spores

**Figure 4:**
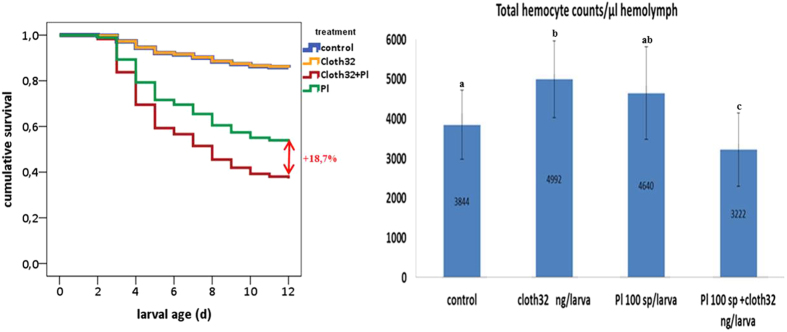
Effects of clothianidin on larval mortality; control: n = 144; cloth32: n = 144; *P. larvae* sp: n = 239; *P. larvae* sp + cloth32: n = 144; and on THC; control: n = 34; cloth32: n = 33; *P. larvae* sp: n = 30; *P. larvae* sp + cloth32: n = 30 (*). Notice the bias caused by the selection of only fully developed and asymptomatic surviving larvae in all groups for hemocyte counts. Hemocyte levels of larvae with symptoms of infection and/or intoxication are not shown here. Error bars represent standard deviation.

A Cox regression analysis showed significant differences in the mortality rate of larvae regarding treatment (see Table 3 for statistics, Fig. 4 for survival curves). Rearing mortality in the control group was 16.7% at the end of the experiment. Feeding a sublethal dose of cloth resulted in 13.9% larval mortality and feeding of *P. larvae* spores resulted in 45.2% (results previously shown). Larvae co-exposed to both stressors showed a significantly higher mortality than the sum of the individual effects, resulting in a mortality of 63.9% at d12. The higher mortality found in this group indicates a synergistic effect between clothianidin and a bacterial infection with *P. larvae*. Similarly, THC and DHC are also affected by the combination of both stressors (see Fig. 4 for THC, Fig. 2 for DHC, Table 2 for statistics). Feeding cloth32 to larvae produces an increase in THC and feeding *P. larvae* spores to larvae leads to an increase in DHC when compared to naïve larvae (results previously shown). Both stressors applied individually increase the cellular response but when comparing this effect against the effect they cause together in the cellular response, a statistically significant reduction in THC and DHC is found in larvae fed on *P. larvae* spores + cloth32 compared to larvae fed on cloth32 or *P. larvae* spores solely. Among differential hemocytes, levels of plasmatocytes and granulocytes were significantly lower in larvae fed on *P. larvae* spores + cloth32 compared to levels in larvae fed on *P. larvae* spores or cloth32. Together, these results suggest that the combined exposure of larvae to a sublethal dose of clothianidin and *P. larvae* spores increases the risk of larval mortality by 18.7% (this is the difference between the mortality caused by *P. larvae* and *P. larvae* + cloth32 after subtracting control mortality), which also correlates with a significant immune depletion of the cellular response in the same larvae.

### Combined effects of fluvalinate and *P. larvae* spores

**Figure 5:**
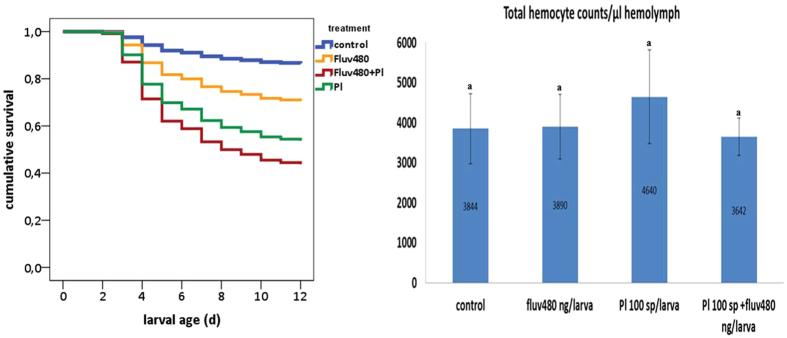
Effects of fluvalinate on larval mortality; control: n = 144; fluv480: n = 144; *P. larvae* sp: n = 239; *P. larvae* sp + fluv480: n = 32; n = 144; and on THC; control: n = 34; fluv480: n = 32; *P. larvae* sp: n = 30; *P. larvae* sp + fluv480: n = 32 (*). Notice the bias caused by the selection of only fully developed and asymptomatic surviving larvae in all groups for hemocyte counts. Hemocyte levels of larvae with symptoms of infection and/or intoxication are not shown here. Error bars represent standard deviation.

A Cox regression analysis showed significant differences in the mortality rate of larvae regarding treatment (see Table 3 for statistics, Fig. 5 for survival curves). Feeding fluv480 resulted in 29.9% of larval mortality (LD_30_), feeding of *P. larvae* spores resulted in 45.2% larval mortality (results previously presented), and basal rearing mortality in the control group was 16.7%. When larvae were co-exposed, mortality at d12 (**38.2**% *-subtracting 16.7 to 54.9*) was similar to the sum of the individual effects **(41.7%)** of fluvalinate (**13.2%**–*subtracting 16.7 to 29.9*) and *P. larvae (**28.5%-**subtracting 16.7 to 45.2*), showing an additive effect on larval mortality. Regarding hemocyte counts, THC and DHC are not affected by the combination of both stressors (see Fig. 5 for THC, Fig. 2 for DHC, Table 2 for statistics). Feeding *P. larvae* spores to larvae leads to an increase of DHC but a single treatment of fluv480 does not alter THC or DHC when compared to naïve larvae (results previously shown). When comparing their individual effect against the effect they cause in the cellular response in combination, no statistically significant reduction is found for THC or DHC between larvae fed on *P. larvae* spores + fluv480 and larvae fed on *P. larvae* spores or fluv480. Among differential hemocytes, levels of plasmatocytes and granulocytes did not differ significantly in larvae fed on *P. larvae* spores + fluv480 compared to larvae fed on *P. larvae* spores or fluv480. Taken together, these results suggest that whereas exposure of larvae to fluvalinate and *P. larvae* spores in combination has an additive effect on larval mortality, it does not alter the cellular response in the same manner as for *P. larvae* spores + dim120 and *P. larvae* spores + cloth32.

### Assessing larval weight

A Krustal Wallis Test followed by a Mann-Whitney U-Test was conducted to account for differences in larval weight among experimental groups. No statistically significant differences were found between control larvae (n = 42) and dim120 larvae (n = 45) or cloth32 larvae (n = 38) (Mann-Whitney U-Test *p* > *0.05*). This result excludes any weight effect of a sublethal dose of dim or cloth in larval development until day 7. Mann-Whitney U-Test showed no statistically significant differences between control larvae and any of the experimental groups (*p* > *0.05*) (see S. Fig. 2), indicating that larvae used for hemocytes counts were all similar in size to the control group.

## Discussion

Bee losses worldwide have been attributed to multiple causes involving complex interactions between pesticides, pathogens and reduction of the natural habitat^5^. Thereby, understanding these interactions is of most importance in order to develop mitigation programs that might reduce the impact of environmental challenges on honeybee health.

Here, we address for the first time a synergistic interaction between a honeybee brood affecting bacterium and different classes of pesticides. Either the organophosphate dimethoate or the neonicotinoid clothianidin fed in sublethal doses to larvae previously infected with AFB significantly elevate larval mortality. These results also correlate with a significant depletion of the cellular immune response in larvae co-exposed to both stressors compared to the effect they cause individually. Similarly, the adverse effects of two neonicotinoids (thiamethoxam and clothianidin) in combination with a gut infection were demonstrated in bumble bees^52^. We also show that feeding a lethal dose of the pyrethroid fluvalinate (~LD_30_) to infected larvae has solely an additive effect to the mortality caused only by the bacteria and not pointing to a synergistic effect. Besides, the cellular response (hemocyte levels) is not altered in these larvae.

In general, previous authors’ works mostly addresses interactions between insecticides in combination with *Nosema* infection in honeybee workers. Alaux *et al*. found a synergistic effect between imidacloprid and *Nosema* that renders the colony more susceptible to the parasite^18^. Similarly, thiacloprid has been related with higher mortality rates of individuals when infections of black queen cell virus (BQCV) or *Nosema ceranae* took place at the same time^21^. Insecticides like fipronil have also been proven to act synergistically with *Nosema ceranae* to elevate adult honeybee mortality^53^. Other authors showed a positive correlation between the consumption of contaminated pollen by honeybee colonies and an increased probability of *Nosema* infection^40^. In other investigations, the interactions thiacloprid-fipronil or imidacloprid-fipronil caused the suppression of immune-related genes and induced higher mortality rates when combined with *Nosema* infection^19^,^53^.

Reports on the effect of pesticides, infections or a combination of both on honeybee larvae are scattered in the literature. Gregorc *et al*. exposed larvae to pesticides and/or *Varroa* mites and found altered transcript levels of several key genes involved in larval immunity, development and detoxification mechanisms^54^. Interestingly, fluvalinate did not have a strong effect in changing the expression of immune- or metabolism-related genes as found for imidacloprid or fungicides. This result is also supported by a previous work that tested the effects of different acaricides commonly used for *Varroa* control on gene expression and pathogen loads in honeybees^55^. Whereas pesticides like coumaphos (organophosphate) altered metabolic processes involving detoxification mechanisms and activation of the cellular response, the gene expression of bees exposed to fluvalinate did not differ from controls. In a different investigation, exposure of honeybee larvae to imidacloprid produced higher transcript levels of genes encoding detoxifying enzymes and lower transcript levels of the gene Hsp90, the latter suggesting a reduced robustness of the larval developmental program^56^. In a recent study, the exposure of honeybees to neonicotinoids similarly triggered the activation of detoxification mechanisms and increased energy demand to support the detoxification processes initiated^57^. Another work has also shown that pesticides such as neonicotinoids increase levels of carboxylesterases and glutathione S-transferases (key detoxification enzymes of pesticides) as well as levels of polyphenol oxidase (PPO) in adult honeybees^58^. Consequently, the latest studies have demonstrated that honeybees exposed to different classes of pesticides undergo molecular changes that trigger activation of signaling pathways involved in larval development, immune responses, detoxification processes and oxidative stress.

American foulbrood infections have also effects on the expression of genes involved in immunity, larval health and stress responses in honeybee larvae. Infection of larvae with *P. larvae* increased the regulation of immune-related genes involved in the production of antimicrobial peptides (AMPs), among others^59^. In-sights into the pathogenesis of *Paenibacillus larvae* revealed differences regarding genotype. When *P. larvae* spores germinate and proliferate in the midgut, strains of ERIC I locate in the middle of the gut lumen from where they produce toxins to disrupt the epithelial tissue. ERIC II (used in this study), however, was shown to require close contact to the epithelium, penetrate it by the paracellular route (as for ERIC I) so it can be further detected between the epithelial and the underlying smooth muscle cell layer^60^. This situation will presumably allow the immune response of the larvae not only to produce AMPs to fight *P. larvae* bacteria, but to carry out processes of phagocytosis and nodulation mediated by hemocytes. Honeybee worker larvae have already been shown to rely on these responses accompanied with humoral responses to clear bacterial infections^61^.

The cellular response of honeybees plays a crucial role in the metabolism of insecticides and is also involved in processes such as phagocytosis, nodulation and encapsulation during infections^17^. Total hemocytes and differential hemocytes are known to increase with both detoxification of insecticides and activation of an immune response following infections, with granulocytes particularly responsible for these two processes^17^,^62^,^63^. Investigations in *Apis mellifera* are pointing to the cellular immune competence as a key parameter to study stressors that bees might confront and to colony health^64^,^65^. In the case of honeybee larvae this is of special interest considering that larvae and pupae have the highest levels of THC, which are decreasing in adult bees while aging^65^. A recent investigation has also demonstrated compromised cellular response in honeybee workers upon exposure to sublethal doses of three neonicotinoids^66^.

The results obtained in our investigations at the phenotypic level complement those results found at the molecular level in the above mentioned literature reporting that fluvalinate does not alter metabolic processes as strongly as other pesticides such as dimethoate or clothianidin. On the one side, we show that larvae exposed to *P. larvae* increase levels of DHC (mostly granulocytes), reflecting the activation of an immune response to an underlying bacterial infection. On the other side, sublethal doses of dimethoate or clothianidin initiated a cellular response and increased levels of THC and DHC compared to controls. Similarly, among differential hemocytes, levels of granulocytes were also significantly higher, reflecting what appears to be a recruitment of hemocytes specialized in detoxification mechanisms. Nevertheless, dimethoate at LD_50_ produced a significant reduction of THC and DHC as compared to control larvae, probably due to the killing of the cells. These effects were not observed when feeding ~LD_30_ or ~LD_60_ fluvalinate. Submitting larvae to fluvalinate did alter neither THC nor DHC, and granulocyte levels did not significantly differ from granulocyte levels found in control larvae. This correlates very well with above mentioned literature reporting none to very low effects of fluvalinate in metabolic processes. Here, we demonstrate that *P. larvae* bacteria, dimethoate or clothianidin at sublethal doses act individually in larvae to activate the cellular response by recruiting hemocytes, especially granulocytes. When larvae are co-exposed to *P. larvae* and dimethoate or clothianidin, a significant reduction of THC and DHC compared to the effect that causes each stressor individually in the cellular response is observed, showing that the cellular response is overwhelmed in co-exposed larvae and demonstrating the double function of hemocytes (and especially granulocytes) to carry out detoxification processes and bacterial clearance. Besides, the mortality rate of co-exposed larvae is significantly higher than the mortality the two stressors cause separately, displaying a synergistic interaction between a bacterial infection and a pesticide. Intriguingly, THC and DHC do not differ significantly in larvae fed with *P. larvae* and fluvalinate compared to the effect each one has individually. In this case, the cellular response is required for bacterial clearance, however apparently not for detoxification of fluvalinate. Besides, larval mortality is in the same range as the sum of the mortalities that each challenge causes separately, indicating an additive effect.

It has been recently illustrated that neonicotinoids like clothianidin and imidacloprid adversely modulate the transcription factor NF-κB, which plays a key role in immunity, eventually affecting immune defenses by reducing the expression of AMPs^67^. Apparently, this is achieved by inhibiting cytokine-producing immune cells that are expressing nicotinic acetylcholine receptors (nAChR). Similarly, a case of synergistic interaction between *Varroa* mite infestation and deformed wing virus (DWV) was associated with colony losses and an immunosuppression characterized also by a down-regulation of NF-κB^22^. The authors claim that the competitive utilization of this transcription factor by DWV and any other stressor (here, by wounding when *Varroa* feeds) promotes a rapid viral replication and killing of bees. The humoral response of honeybees is mediated by four immune pathways: Toll, Imd, Jnk and Jak/Stat^68^. Toll and Imd are NF-κB-like signaling pathways involved in the regulation of genes coding antimicrobial peptides. Since neonicotinoids interfere with the immune response by reducing levels of NF-kB, it seems that in our investigations a sublethal dose of clothianidin renders individuals more susceptible to bacterial infection. Larvae which are co-exposed to *P. larvae* and clothianidin show reduced levels of total and differential hemocytes, clothianidin acts negatively in the regulation of NF-κB and, eventually elevates the risk of mortality when NF-κB is a key element for production of AMPs during infection with AFB. Similar results could be expected for neonicotinoids classified as *N*-nitroguanidines (imidacloprid, thiamethoxam, clothianidin and dinotefuran). Organophosphates are inhibitors of acetylcholinesterase (AChE), which might explain the similar result obtained when we fed larvae with dimethoate but not fluvalinate^6^,^69^. It is also known that organophosphates have an impact on the number of hemocytes, differentiation and phagocytosis^17^. Our results demonstrate that granulocytes are involved in both elimination of toxins and fighting of bacterial infections, which could cause a competitive need for hemocytes when required in both functions. These results could also be of interest during winter, since it has been demonstrated that winter bees may undergo bacterial infections that compromise their cellular response^70^. If honeybees feed on honey and pollen stores containing pesticides during winter and besides, suffer from persistent bacterial infections, the consequences for colony health might be more devastating than previously thought.

Finally, it is of particular importance to highlight that all three pesticides used in this work caused a significant increase in the levels of oenocytoids compared to control larvae. Oenocytoids are the primary producers of cuticular hydrocarbons, which are involved in chemical communication between nest-mates^71^. The last authors found that worker bees are capable of recognizing infected nest-mates and display behavioral changes such as excessive grooming, aggression behavior or removal of infected individuals. These changes in behavior are triggered by the different composition of the hydrocarbon bouquet present in the cuticula of infected bees compared to naïve bees. Thus, honeybee nurses in colonies infected with AFB are able to detect diseased larvae and cannibalize them due to likely changes in the composition of the hydrocarbon profile^50^. In our investigations, larvae infected with *P. larvae* presented elevated levels of oenocytoids, which might account for differences in the composition of the hydrocarbons on their surface and would allow nurse bees to detect and eliminate compromised larvae in natural conditions. Such elevated levels of oenocytoids were also found for larvae treated with dimethoate, clothianidin and fluvalinate. This opens the question whether pesticides could indirectly alter the hydrocarbon composition of larvae and may therefore, have far more implications for colony health than previously thought. Whether pesticides change chemical communication in a colony and, in consequence, initiate an active removal of intoxicated larvae seems to be of most interest for future research.

## Methods

### Honeybees, chemicals and spore suspensions of *P. larvae*

All honeybees used in this study belong to *Apis mellifera* subsp*. carnica* and were kept in the garden apiary of the Karl-Franzens University of Graz under normal living conditions. Colonies are regularly treated against *Varroa* according to standard practices. No apparent symptoms of *Varroa* infestations and/or virus infections were observed by the time experiments were carried out.

Three different classes of chemicals were used as stressors during experiments, the organophosphate dimethoate (*dim* at three doses; 120, 240 and 360 ng/larva; total amount given during artificial rearing), the neonicotinoid clothianidin (*cloth* at three doses; 8, 16 and 32 ng/larva), both highly toxic to bees, and the pyrethroid fluvalinate (*fluv* at three doses; 240, 480 and 720 ng/larva), considered to have a low toxicity to bees and commonly used for control of *Varroa* infestations in honey bee colonies. All chemicals were purchased at *LGC Standards GmbH Dr. Ehrenstorfer* (Wesel, Germany). Stock solutions of pesticides were prepared in acetone (1 mg/ml), subsequent dilutions were done in deionized water, and acetone concentrations never exceeded 1% of the larval food.

To carry out bacterial infection of honeybee larvae we used spores of *Paenibacillus larvae (P. larvae*) (strain 233/00) genotype Eric II, due to its high virulence at the individual level^55^. To obtain spores of *P. larvae*, a few colony forming units (CFU) of *P. larvae* Eric II were used to inoculate MYPGP-agar slants and incubated at 34.5 °C for 12 to 14 d. Subsequently, the liquid supernatant was collected, heated for 10 min at 85 °C to eliminate vegetative forms (three repetitions with each 10 min at room temperature between treatments) and used to determine spore concentration by cultivating serial dilutions on MYPGP-agar plates. The spore suspension was stored at 4 °C and used throughout all experiments.

### Larval rearing to assess mortality due to a pesticide, bacterial infection or their combination

Honeybee larvae were reared in 48 well plates according to a method described by Aupinel *et al*.^72^, which was further developed in our laboratory^73^. One plate with 48 larvae from three different colonies (16 larvae/colony – in order to equal out differences between colonies) was considered as one experimental replicate and a minimum of three replicates for each experimental group were carried out on different days. During grafting, first instar worker larvae (*ca* 5–10 h old) were assigned to one of the following experimental groups; control larvae fed on regular diet, larvae fed on diet containing one of the three pesticides at one concentration, larvae fed on diet containing *ca* 100 spores of *P. larvae*, and larvae fed on diet containing *ca* 100 spores of *P. larvae* and one pesticide (see S. Table 1). A control group of larvae fed on regular diet + acetone (1%) was also included. Mortality rates were recorded daily during the following 12 d. Larvae feeding on contaminated diet were grafted in 10 μl of regular larval diet and another 10 μl of contaminated larval diet were added after grafting, reaching a total amount of 20 μl of diet on the first rearing day. On the second day, feeding was paused (for all treatment groups) to allow larvae feeding on contaminated diet to consume the total amount of food and therefore, guarantee the complete ingestion of spores within the first 48 h of life. In the case of larvae feeding on pesticides, chemicals were added on days 3, 4, 5 and 6 in order to simulate chronic exposure and reach final doses on day 6.

To confirm the inoculum of *ca* 100 spores, contaminated diet fed to larvae was plated for every single experiment on MYPGP-agar and CFU were counted six days later. Larval diet with a 50% content of royal jelly has a strong antibacterial activity. Plating diet on agar allows spores to germinate and CFU appear after 3–6 d. Germination of *P. larvae* spores was confirmed when no bacterial growth was detected before day 3, which indicated that no significant bacterial contamination was present.

### Assessing hemocyte counts in larvae

Larvae were selected to determine larval weight before assessing hemocyte levels. In the case of the two sublethal concentrations of pesticides used (dim120 and cloth32), differences in larval weight compared to the control larvae could be indicative of a delayed larval development. Nevertheless, for all other groups lethal doses were used, only larvae that developed as well as controls were taken for hemocyte counts, i.e. weight was used to normalize the size among larvae. The immune competence of larvae was studied by counting total hemocytes (THC) and differential hemocytes (DHC) at day 7 of larval development. These groups of larvae consisted in replicates of the same groups destined to assess larval mortality and were always reared in parallel. Here we refer to differential hemocytes to all other hemocyte types observed, which are no longer in the prohemocyte stadium, but have reached a different developmental stadium (granulocyte, oenocytoid or plasmatocyte). Total hemocyte counts include prohemocytes and all differential hemocytes observed. Hemolymph samples were extracted by puncturing larvae with a sterile needle and subsequent collection of 1 μl of hemolymph by using a sterile glass micro capillary. A Bürker-Türk hemocytometer was used to determine hemocyte counts in a 1/10 dilution. Differential hemocytes were identified by their morphology as described in the literature^63^,^74^ and by using own pictures (see Pic. 1, Supplementary Material).

### Final experimental groups to assess individual or combined effect of the tested stressors were as follows

#### Individual effect of pesticides or bacterial infection on larval mortality and hemocyte counts

Control larvae, control acetone (1%), dim120 ng/larva, dim240 ng/larva, dim360 ng/larva, cloth8 ng/larva, cloth16 ng/larva, cloth32 ng/larva, fluv240 ng/larva, fluv480 ng/larva, fluv720 ng/larva, and *ca* 100* P. larvae* spores.

#### Combined effect of pesticides and bacterial infection on larval mortality and hemocyte counts

Control larvae, control acetone (1%), ~100 *P. larvae* spores, dim120 ng/larva + 100* P. larvae* spores, cloth32 ng/larvae + 100* P. larvae* spores, and fluv480 ng/larvae + 100* P. larvae* spores. One concentration of each pesticide (sublethal for dim and cloth, ~LD_30_ for fluv) was chosen to feed larvae with one pesticide and *ca* 100 spores of *P. larvae*. Since fluvalinate is considered non-toxic to bees in the concentrations applied for *Varroa* treatment, we decided to work with the ~LD_30_ in order to magnify possible effects on larval mortality or hemocyte levels when applied in combination with *P. larvae* spores.

### Statistical analysis

The software package SPSS v. 19 was used for statistical analyses. A Cox regression analysis was performed to estimate differences in larval mortality among treatments. Regarding levels of hemocytes, an examination of the histograms of the distribution of total hemocyte counts and differential hemocyte counts showed a deviation from normal distribution as shown in other investigations^65^,^66^. Therefore, non-parametric methods (Mann-Whitney U-Test/Kruskall Wallis) were used for statistical analysis. Non-parametric methods were also carried out to assess differences in larval weight among treatments.

## Additional Information

**How to cite this article**: López, J. H. *et al*. Sublethal pesticide doses negatively affect survival and the cellular responses in American foulbrood-infected honeybee larvae. *Sci. Rep.* **7**, 40853; doi: 10.1038/srep40853 (2017).

**Publisher's note:** Springer Nature remains neutral with regard to jurisdictional claims in published maps and institutional affiliations.

## Supplementary Material

Supporting Information

## Figures and Tables

**Table 1:** Results of the Cox regression analysis for mortality of larvae feeding on pesticide or *Pl* spores individually.

	Cumulative mortality at d12 (%)	Wald	df	*p-value*	Exp(B)	95,0% CI for Exp(B)	
						Lower	Upper
**control**	**14.58**						
*control acetone	**16.32**						
**treatment**		170.39	4	***0.000***			
acetone	*	0.472	1	0.492	1.158	0.762	1.758
**dim 120**	**14.58**	0.001	1	***0.972***	1.008	0.634	1.604
replicates		4.958	3	0.175			
**dim 240**	**47.22**	53.305	1	***0.000***	3.936	2.725	5.686
replicates		5.946	2	0.051			
**dim 360**	**63.89**	102.303	1	***0.000***	6.029	4.256	8.539
replicates		19.057	2	0.000			
replicate (1)		11.843	1	0.001	2.374	1.451	3.883
replicate (2)		0.186	1	0.666	0.884	0.504	1.549
**treatment**		6.398	4	***0.171***			
acetone	*	1.699	1	0.192	1.541	0.804	2.954
**cloth8**	**8.33**	1.108	1	***0.293***	0.616	0.250	1.518
**cloth16**	**17.5**	0.572	1	***0.449***	1.352	0.619	2.955
**cloth32**	**13.89**	0.006	1	***0.936***	1.033	0.462	2.312
replicates		1.948	2	0.378			
**treatment**		62.951	4	***0.000***			
acetone	*	0.705	1	0.401	1.278	0.721	2.263
**fluv240**	**20.83**	5.455	1	***0.020***	1.989	1.117	3.541
**fluv480**	**29.86**	15.858	1	***0.000***	3.010	1.750	5.177
**fluv720**	**48.61**	44.837	1	***0.000***	5.949	3.530	10.025
replicates		1.918	2	0.383			
**treatment**							
***Pl*****spores**	**45.19**	37.660	1	***0.000***	3.495	2.344	5.212

When significances in replicates were found for one treatment, details are given to indicate in which specific pesticide concentration significances were found. These significances account for standard variation in rearing practices since not always the same three colonies were used from replicate to replicate. *Final mortality in these experiments.

**Table 2:**
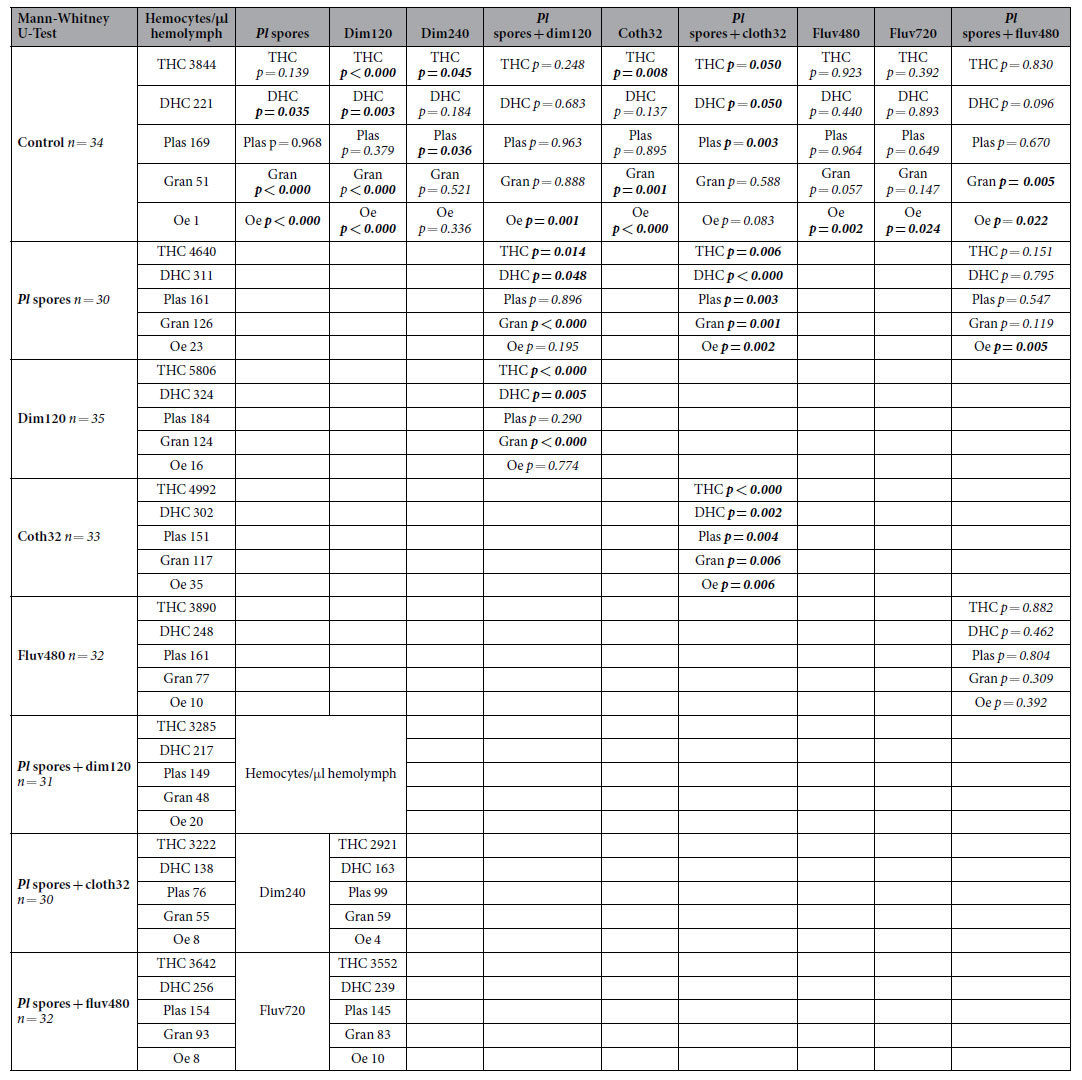
Mann-Whitney U-Test comparisons for total hemocytes counts (THC), differential hemocytes counts (DHC), plasmatocytes (plas), granulocytes (gran) and oenocytoids (oe).

Notice the bias caused by the selection of only fully developed and asymptomatic surviving larvae in all groups. Symptomatic larvae presented none to very low levels of hemocytes and were not used for comparisons. Significances are denoted in bold. Colored letters highlight the comparisons made between the combined effect and the effect of the individual treatment.

**Table 3:** Results of the Cox regression analysis for mortality of larvae feeding on pesticide and *Pl* spores in combination.

	Cumulative mortality at d12 (%)	Wald	df	*p-value*	Exp(B)	95,0% CI for Exp(B)	
						Lower	Upper
**control**	**16.67**						
*Pl spores	**45.19**						
**treatment**		88.040	3	***0.000***			
** dim120**	**14.58**	0.660	1	***0.417***	0.773	0.415	1.439
** *****Pl*** **spores +dim120**	**59.03**	30.799	1	***0.000***	4.138	2.506	6.834
** *****Pl*** **spores**	*	37.334	1	***0.000***	3.478	2.332	5.187
replicates		4.223	3	0.238			
***Pl*** **spores +dim120 &** ***Pl*** **spores**	**—**	15.043	—	***0.000***	0.524	0.378	0.727
**treatment**		66.108	3	***0.000***			
** cloth32**	**13.89**	0.287	1	***0.592***	0.858	0.489	1.505
** *****Pl*** **spores +cloth32**	**63.89**	23.538	1	***0.000***	3.743	2.196	6.380
** *****Pl*** **spores**	*	38.773	1	***0.000***	3.562	2.388	5.312
replicates		7.049	2	0.029			
replicate (1)		6.989	1	0.008	0.502	0.301	0.837
replicate (2)		0.754	1	0.385	0.809	0.501	1.306
***Pl*** **spores +cloth32 &** ***Pl*** **spores**	—	17.962	—	***0.000***	0.504	0.367	0.692
**treatment**		41.504	3	***0.000***			
** fluv480**	**29.86**	8.449	1	***0.004***	1.984	1.250	3.148
** *****Pl*** **spores +fluv480**	**54.86**	13.900	1	***0.000***	2.965	1.674	5.250
** *****Pl*** **spores**	*	38.155	1	***0.000***	3.525	2.364	5.258
replicates		7.307	2	0.026			
replicate (1)		3.194	1	0.074	0.586	0.326	1.053
replicate (2)		0.872	1	0.351	1.274	0.766	2.120

When significances in replicates, they account for standard variation in rearing practices. Not always the same three colonies were used from replicate to replicate.
