# Supplementary material for: Sublethal pesticide doses negatively affect survival and the cellular responses in American foulbrood-infected honeybee larvae
Source: Sci Rep. 2017 Feb 1;7:40853. doi: 10.1038/srep40853 (PMC5286422; doi:10.1038/srep40853)
Supplement: Supporting Information [file srep40853-s1.doc]

**Supporting information**

**Sublethal pesticide doses negatively affect survival and the cellular responses in American foulbrood-infected honeybee larvae**

**Authors:** Javier Hernández López*, Sophie Krainer, Antonia Engert, Wolfgang Schuehly, Ulrike Riessberger-Gallé, Karl Crailsheim

**
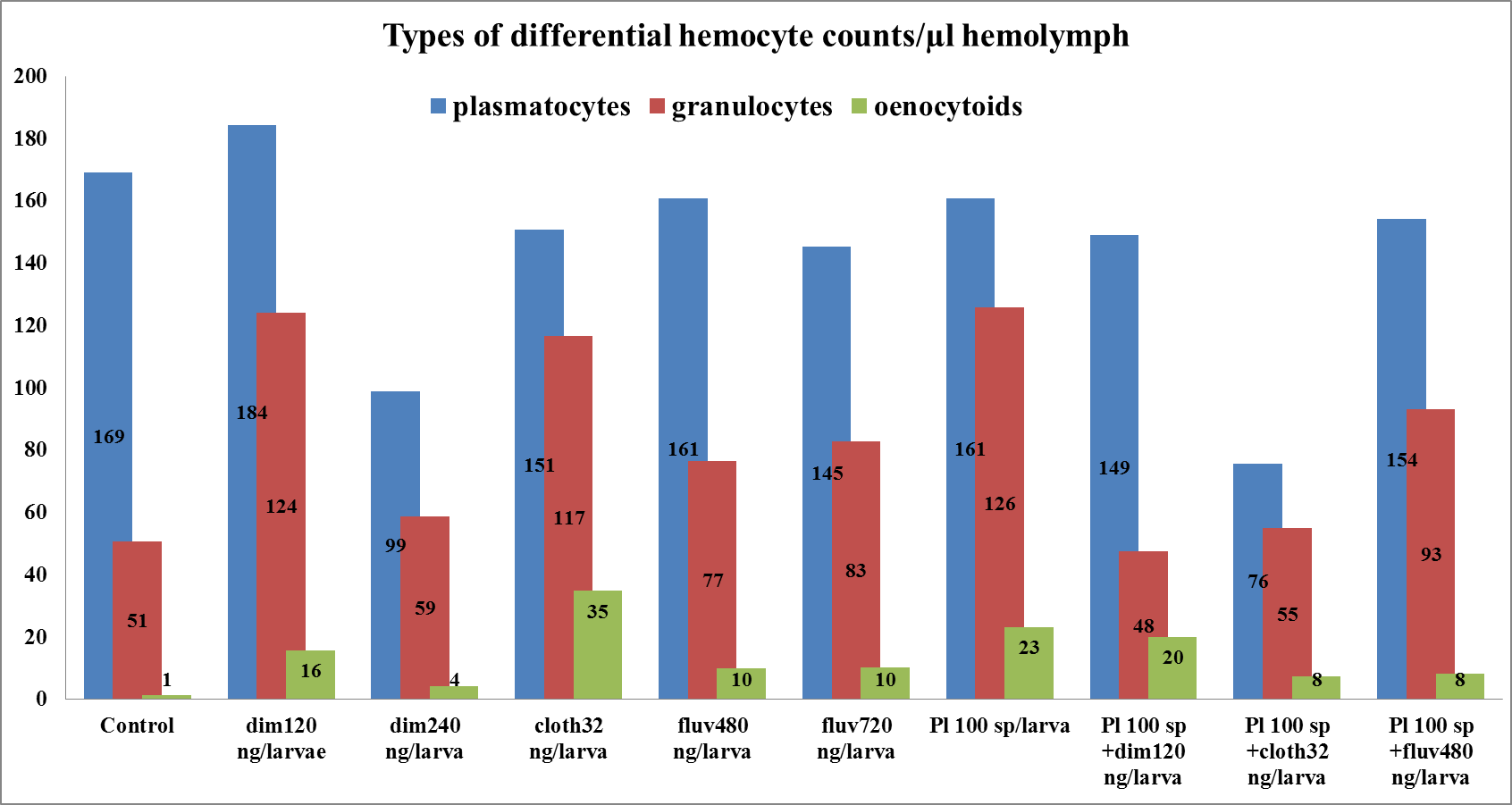
**

**S.Fig.1.** Changes in the no. of differential hemocytes types regarding feeding regime of honey bee larvae. Control: n=34; dim120: n=35; dim240: n=23; cloth32: n=33; fluv480: n=32; fluv720: n=22; *Pl* sp: n=30; *Pl* sp +dim120: n=31; *Pl* sp +cloth32: n=30; *Pl* sp +fluv480: n=32.


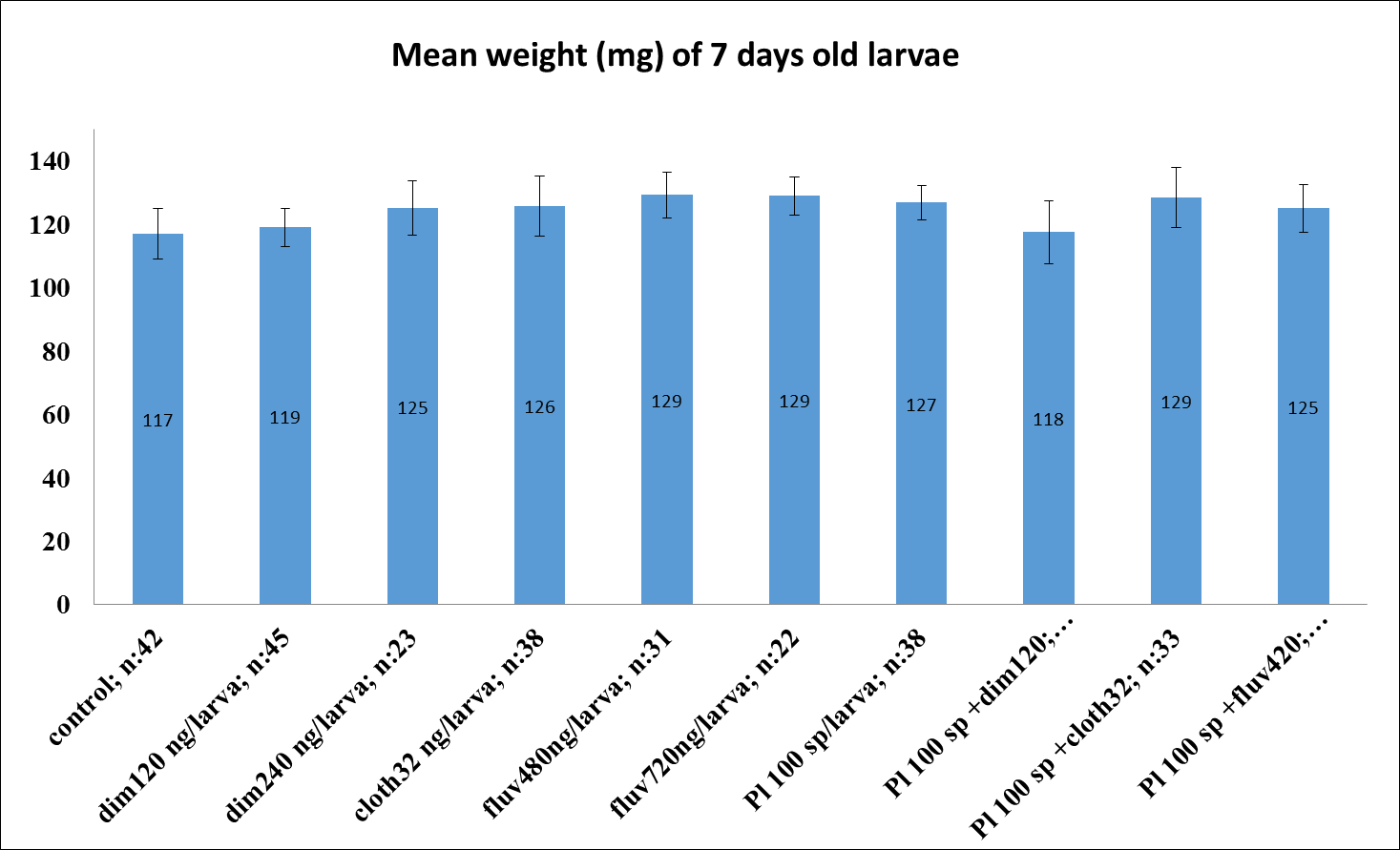


**S.Fig.2.** Mean weight of larvae among experimental groups. Error bars represent standard deviation.


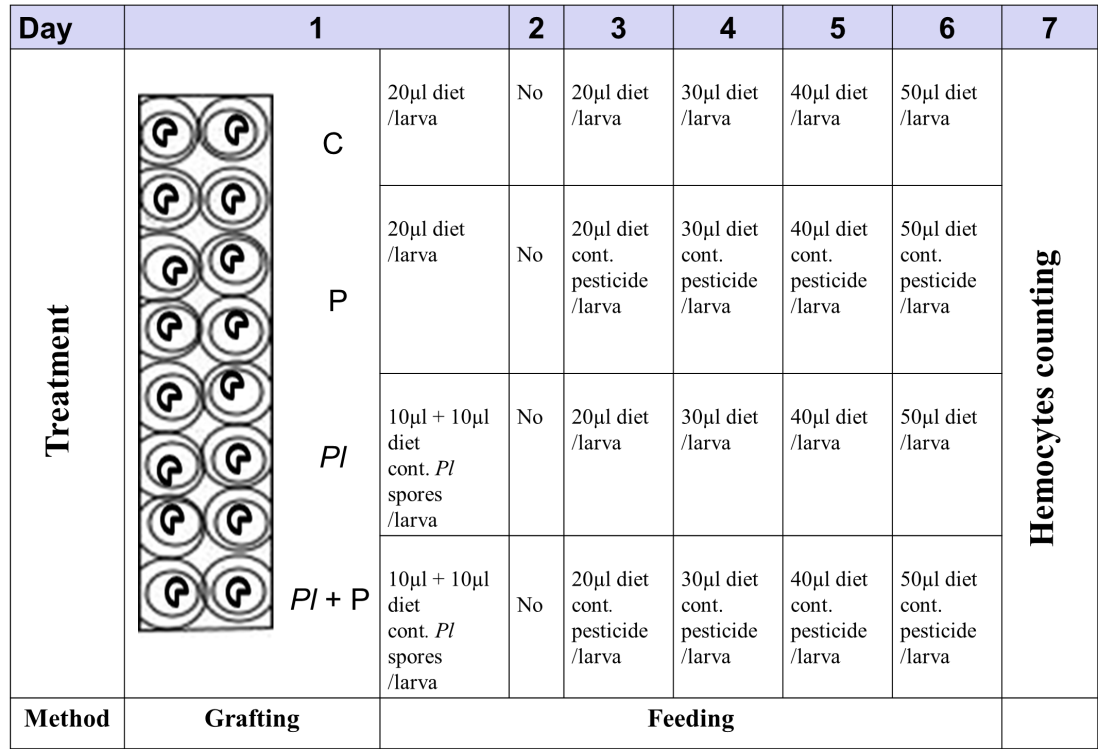


**S.Table1.** Experimental setup of larval rearing groups. C: control, P: pesticide, *Pl*: *Paenibacillus larvae.*


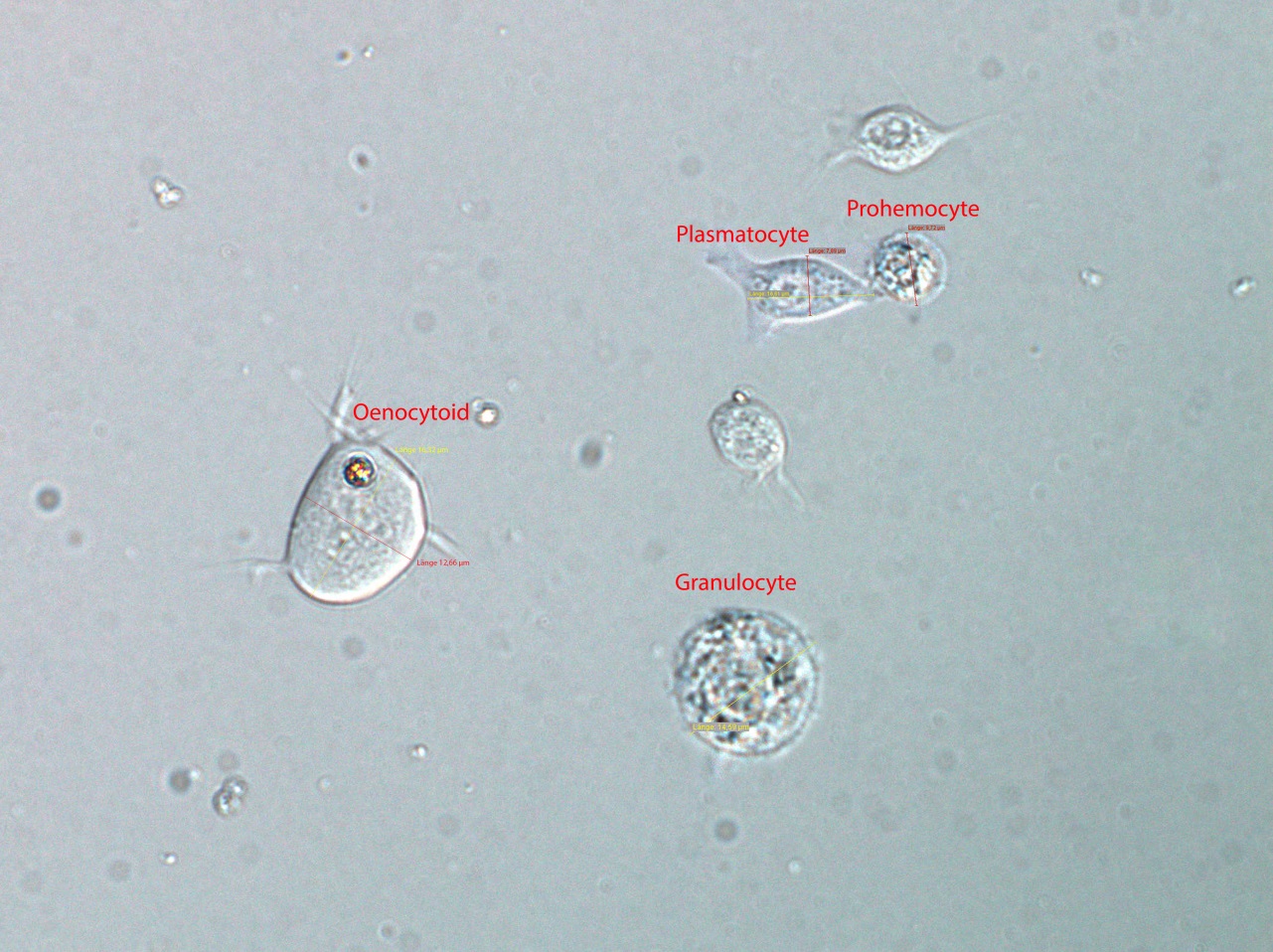


**Pic1.** Hemocyte types of honeybee larvae. Photo composed from two original images. DIC microscopy
